# Kinetic surface roughening and wafer bow control in heteroepitaxial growth of 3C-SiC on Si(111) substrates

**DOI:** 10.1038/srep15423

**Published:** 2015-10-21

**Authors:** Li Wang, Glenn Walker, Jessica Chai, Alan Iacopi, Alanna Fernandes, Sima Dimitrijev

**Affiliations:** 1 Queensland Micro- and Nanotechnology Centre, Griffith University, Nathan, QLD, 4111, Australia; 2 Bluglass Ltd., 74 Asquith Street, Silverwater, NSW, 2128, Australia

## Abstract

A thin, chemically inert 3C-SiC layer between GaN and Si helps not only to avoid the “melt-back” effect, but also to inhibit the crack generation in the grown GaN layers. The quality of GaN layer is heavily dependent on the unique properties of the available 3C-SiC/Si templates. In this paper, the parameters influencing the roughness, crystalline quality, and wafer bow are investigated and engineered to obtain high quality, low roughness 3C-SiC/Si templates suitable for subsequent GaN growth and device processing. Kinetic surface roughening and SiC growth mechanisms, which depend on both deposition temperature and off-cut angle, are reported for heteroepitaxial growth of 3C-SiC on Si substrates. The narrower terrace width on 4° off-axis Si enhances the step-flow growth at 1200 °C, with the roughness of 3C-SiC remaining constant with increasing thickness, corresponding to a scaling exponent of zero. Crack-free 3C-SiC grown on 150-mm Si substrate with a wafer bow of less than 20 μm was achieved. Both concave and convex wafer bow can be obtained by *in situ* tuning of the deposited SiC layer thicknesses. The 3C-SiC grown on off-axis Si, compared to that grown on on-axis Si, has lower surface roughness, better crystallinity, and smaller bow magnitude.

Cubic silicon carbide (3C-SiC) grown on Si has many applications due to its low cost, chemical inertness, low lattice mismatch to III-nitrides and graphene, large bandgap, and excellent mechanical properties^1^,^2^,^3^,^4^,^5^. For example, the use of a thin, chemically inert SiC buffer layer between GaN and Si can prevent the “melt-back” etching effect, which is often observed for direct GaN on Si deposition^1^; the excellent lattice and thermal match between SiC and GaN has also been shown to result in crack-free GaN films grown on SiC/Si templates^2^; furthermore, AlGaN/GaN high electron mobility transistors^6^ and light-emitting diodes^7^, fabricated on 3C-SiC/Si substrates, have been demonstrated. In addition, the large refractive index difference between SiC and AlN enabled the fabrication of AlN/SiC distributed Bragg reflector (DBR) to enhance the light reflectance for nitrides deposited on Si substrates^8^.

Previous studies have shown that the strain relaxation and dislocation density reduction in the grown GaN/3C-SiC/Si layers are affected by the thickness, roughness, off-cut angle, curvature magnitude and shape of the 3C-SiC/Si template^3^,^9^,^10^,^11^. Both thin (50 nm) and thick (2500 nm) SiC films have been reported to lead to the generation of cracks in GaN layers^2^,^11^, whereas an intermediate thickness of 700 ~ 1000 nm 3C-SiC/Si acted as the best template to suppress crack generation. The roughness of SiC was found to increase with its thickness^9^,^11^,^12^, and that a reduction of surface roughness by mechanical polishing led to an improvement in GaN crystalline quality and a reduced wafer bow^9^. By using SiC grown on 4° off-axis Si substrate, a reduction in the threading dislocation density in GaN was observed^9^. Concave wafer bending in SiC/Si(111) is preferred because it can counteract the convex wafer bending in the grown GaN/AlN/SiC/Si system^9^. Therefore, in order to inhibit crack generation and reduce dislocation density in GaN grown on 3C-SiC/Si templates, the thickness, roughness, off-cut angle, and wafer bow of the SiC/Si template need to be carefully selected. In this paper, the impact of growth temperature and substrate off-cut angle on 3C-SiC roughness, crystalline quality, and wafer bow are investigated. For SiC grown on on-axis Si, the SiC surface roughness is found to increase with thickness. In contrast, SiC deposited on off-axis Si has a constant surface roughness, being independent of film thickness, consistent with what is expected for a step-flow growth mode. The growth and roughening mechanisms for 3C-SiC are proposed, based on the surface morphology and roughness evolution characteristics. Furthermore, both concave and convex wafer bow can be obtained by adjusting SiC thickness deposited on both sides of a Si wafer.

## Results and Discussions

### Kinetic roughening of grown SiC film.

According to the kinetic roughening theory, RMS roughness *ω* increases as a power law with growth time:


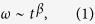


where *t* is growth time and *β* is scaling exponent^13^, which is a measure of how fast the surface roughness develops. A high *β* value is indicative of the dominance of three-dimensional (3D) growth modes, resulting in high roughness films for thick films. A low *β* value indicates that there is a competition between two-dimensional (2D) and 3D growth modes, with the 2D growth mode proportionally more dominant for lower *β* values. True layer-by-layer and step-flow growth mode occur for a *β* value of zero, indicating that roughness is independent of film thicknesses. Because the SiC thickness is linearly proportional to growth time, therefore, one could replace time *t* by thickness *h* in equation 1:


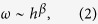


**Figure 1:**
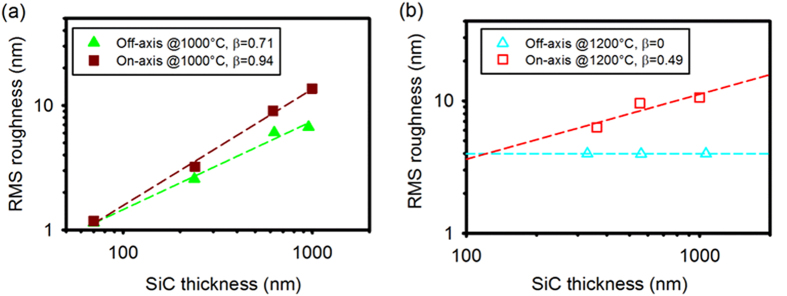
The log-log scale plots of the dependence of RMS roughness on SiC thickness. SiC deposition was performed on both on-axis and 4° off-axis Si(111) substrates at 1000 °C (**a**) and at 1200 °C (**b**). Scaling exponent has been determined using linear regression. Zero value for the kinetic scaling exponent was found for SiC ranging from ~300 nm to ~1100 nm grown on off-axis Si at 1200 °C. Before the 1200 °C deposition, ~100 nm SiC was initially grown at 1000 °C to inhibit the formation of Si voids at the 3C-SiC/Si interfaces. The ~100 nm SiC/Si has a roughness of ~3.61 nm and ~3.81 nm for on-axis and off-axis SiC/Si, respectively, before the growth commenced at 1200 °C.

The root mean square (RMS) roughness of grown 3C-SiC was measured in a 5 μm × 5 μm area using non-contact atomic-force microscope (AFM). The dependence of SiC RMS roughness on thickness is shown in Fig. 1(a,b), in log-log scale, and the scaling exponent was calculated by linear regression. A sharp increase in roughness with SiC thickness was seen for SiC grown at 1000 °C, with a smaller scaling exponent being observed for off-axis wafer (*β* = 0.71) compared to that for on-axis wafer (*β* = 0.94).

The two most important kinetic energy parameters involved in epitaxial growth are surface diffusion barrier and the Ehrlich-Schwoebel (ES) step-edge barrier (deposition of surface adatoms at the hills of the rough surface because of the presence of step-down barrier)^14^. In the chemical vapour deposition process, diffusion dominated surface smoothing process is competing with roughening process caused by ES step-edge barrier, random ballistic deposition, shadowing effect, and nucleation of three dimensional SiC islands on the terraces^15^,^16^. Due to the relatively shorter diffusion length at the deposition temperature of 1000 °C, the roughening process played a much more significant role, which in turn, led to the roughening of the surface with growth time. Therefore, the surface morphology is dominated by random 3D nucleation centres, as already reported in ref. 12. The denser steps on off-axis Si contributes towards the smoothening process as step-flow growth is enhanced on vicinal substrates, which resulted in a smaller *β* than that of on-axis wafer as shown in Fig. 1(a). When the growth temperature was raised to 1200 °C, the scaling exponent *β* was found to reach approximately zero (shown in Fig. 1(b)) for SiC grown on 4° off-axis Si substrate with a saturated RMS roughness of 3.99 ± 0.2 nm. It is important to note that the majority of this roughness was inherited from the ~100 nm SiC that was deposited at 1000 °C (in the specific case of *β* = 0, the roughness is likely dominated by initial nucleation conditions). When the temperature was raised from 1000 to 1200 °C, the RMS roughness of this ~100 nm SiC was found to increase from less than 2 nm to ~3.61 nm and 3.81 nm for on-axis and off-axis wafers, respectively. In the future, techniques such as adjusting the growth temperature and using thinner SiC films will be explored with the aim of reducing the roughness of this SiC barrier layer.

**Figure 2:**
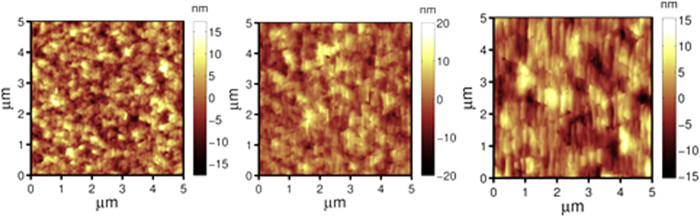
AFM top-view morphology of SiC(111) grown on 4° off-axis Si(111) at 1200 °C with different thicknesses: (**a**) 365 ± 5 nm, (**b**) 556 ± 10 nm, (**c**) 1000 ± 15 nm, scan area is 5 μm × 5 μm, the value of the RMS roughness is constant at 3.99 ± 0.2 nm.

**Figure 3:**
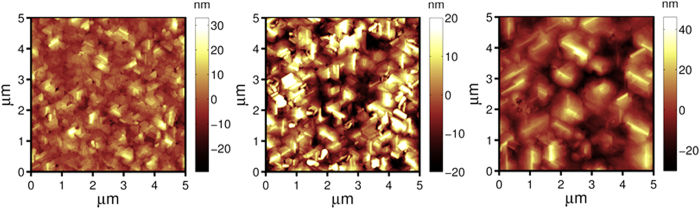
AFM top-view morphology of SiC(111) grown on on-axis Si(111) at 1200 °C with different thicknesses (scan area is 5 μm × 5 μm): (**a**) 365 ± 5 nm (RMS roughness is 6.31 ± 0.3 nm), (**b**) 556 ± 10 nm (RMS roughness is 9.60 ± 0.3 nm), (**c**) 1000 ± 15 nm (RMS roughness is 10.61 ± 0.3 nm).

The AFM top-view surface morphology of SiC grown at 1200 °C is shown in Fig. 2 (for off-axis) and Fig. 3 (for on-axis). Steps parallel to [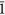
10]/[1
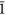
0] direction can be seen in Fig. 2 for off-axis wafers, they indicate that the SiC growth proceeds in a steady step-flow mode, meaning that the adsorped atoms have sufficient diffusion energy to reach the step edges, resulting in well-controlled lateral growth. In addition, the incoming precursor fluxes were also controlled in a condition where 3D nucleation is minimized or inhibited on the terraces. As a comparison, the scaling exponent *β* for on-axis wafers reduced from 0.94 to 0.49 when the growth temperature was raised from 1000 to 1200 °C, yet it still resulted in a significant roughness increase with film thickness (the RMS roughness increased from 6.31 ± 0.2 nm at a thickness of ~362 nm to 9.60 ± 0.3 nm at a thickness of ~550 nm, and to 10.61 ± 0.3 nm at a thickness of ~1000 nm). The domain size (AFM top-view image shown in Fig. 3) increased with thickness, indicating the lateral coalescence of domains, but 3D growth still dominated due to insufficient surface diffusion energy relative to its larger terrace width. The ~550 nm on-axis SiC (RMS roughness of ~9.60 nm) is smoother than the SiC grown at a similar temperature of 1220 °C by another group (RMS roughness of larger than 18 nm for ~500 nm 3C-SiC)^11^, but rougher than the SiC grown at a higher temperature of 1350 °C (RMS roughness of 2.9 nm for ~500 nm)^17^, indicating that the elevated temperature aids the smoothening process during the deposition of SiC.

### Growth models for 3C-SiC deposition on on-axis and off-axis Si substrates

**Figure 4:**
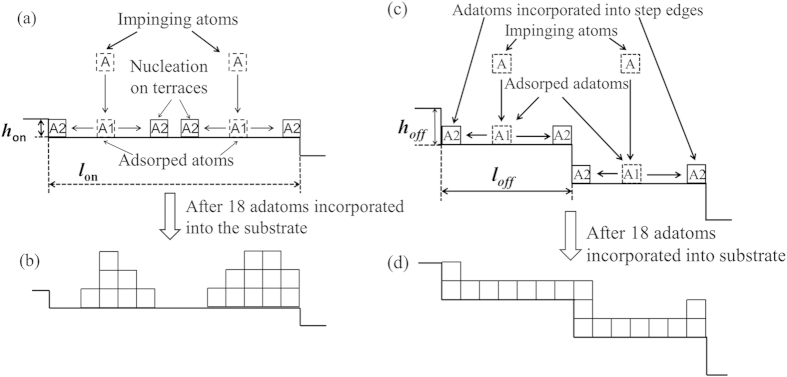
Illustrations of different growth mechanisms for SiC films grown at 1200 °C. On-axis Si, (**a**) surface adatoms diffuse on the surface and form new nucleation centres, leading to wedding-cake type 3D islands as shown in (**b**) after 18 adatoms are incorporated into the Si substrate. For off-axis Si, (**c**) surface adatoms have sufficient energy to diffuse to a step edge, which lead to a constant surface roughness as shown in (**d**) after 18 adatoms are incorporated into the Si substrate.

Based on the roughness evolution and surface morphology seen in SiC film deposited at 1200 °C on substrates with different off-cut angles, growth mechanisms for SiC grown on on-axis and off-axis Si are proposed and illustrated in Fig. 4. In the case of on-axis surface, regular surface steps with height *h*_on_ are separated by the distance *l*_on_, when the impinging atoms are adsorped on the surface, they diffuse either to a step edge or to form 3D nucleation centres on the terraces as illustrated in Fig. 4(a). For example, after depositing 18 atoms, wedding-cake type growth mounds are formed on the surface, as shown in Fig. 4(b), which leads to an increase in overall roughness. As shown in Fig. 4(c), for 4° off-axis Si, it has surface steps with height *h*_off_ which are separated by the equal distance *l*_off_, which is equal or less than half of *l*_on_ (the terrace width of on-axis and off-axis Si was investigated by AFM and the results were published in ref. 12). The required diffusion length is therefore at least halved for an adatom to incorporate into a step edge, and as a result, steady step-flow growth is achieved on off-axis wafer as shown in Fig. 4(d), where the roughness remained constant with increasing thickness.

### Crystallinity analysis

**Figure 5:**
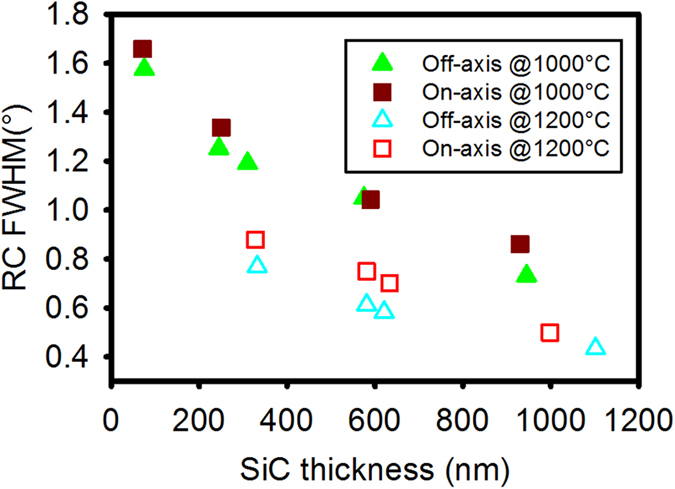
FWHM of HRXRD rocking curve of 3C-SiC(111) peak, SiC films that were grown on both on-axis and 4° off-axis Si(111) substrates at two different growth temperatures.

The crystalline quality of grown SiC was evaluated by the high resolution X-ray diffraction (HRXRD) rocking curve (RC) measurements (shown in Fig. 5). Narrower full-width at half-maximum (FWHM) of 3C-SiC(111) peak was observed for SiC grown on off-axis substrates in comparison to that grown on on-axis substrates at both growth temperatures. A reduction in FWHM with increasing thickness was seen for all films due to the better coalescence and the reduction in defects density, as the broadening of FWHM is mainly caused by domain tilting and presence of defects leading to a change in lattice constant (such as dislocations and stacking faults). The following equation is used to calculate curvature-induced broadening in the FWHM of 3C-SiC(111) peak^18^:


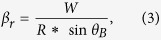


where 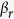 is rocking curve broadening due to curvature of the 3C-SiC/Si wafer, *W* is the width of incident x-ray beam (1mm), *R* is the radius of curvature, and 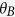 is the Bragg angle of the SiC(111) plane. The calculated curvature-induced broadening is less than 0.01° based on the curvature radius measured by Tencor Flexus 2320 system (will be shown in next section), which is negligible in comparison to the obtained FWHM value (>0.4°). By raising the growth temperature from 1000 °C to 1200 °C, a FWHM reduction of 0.3 ~ 0.4° was seen for ~1000 nm SiC. Better quality was obtained for SiC grown on off-axis Si, with a FWHM of 0.43° (1548 arcsec) at a thickness of 1100 nm. Importantly, this value is comparable to the value reported for SiC grown on 4° off-axis Si at the much higher temperature of 1350 °C^11^. A suitable lower temperature process should suffer less from thermal expansion coefficient mismatch and result in films with lower residual stress and reduced wafer bow, but still deposit films with similar crystal quality. For the ~500 nm film, the observed FWHM for on-axis wafer is 0.71 ± 0.05°, which is wider than what was reported for SiC film grown at 1350 °C^17^. Therefore for SiC deposited on on-axis Si, higher deposition temperatures are required to enhance the adatom diffusion length and to improve both the surface smoothness and the crystal quality. In contrast, the narrower terrace width on off-axis wafer requires relatively smaller diffusion length to achieve step-flow growth, which can be readily achieved at a relatively lower deposition temperature. It has previously been reported that the deposition temperature for homoepitaxial growth of high quality SiC can be reduced by utilizing off-axis substrates^19^.

### Wafer curvature and stress analysis

**Figure 6:**
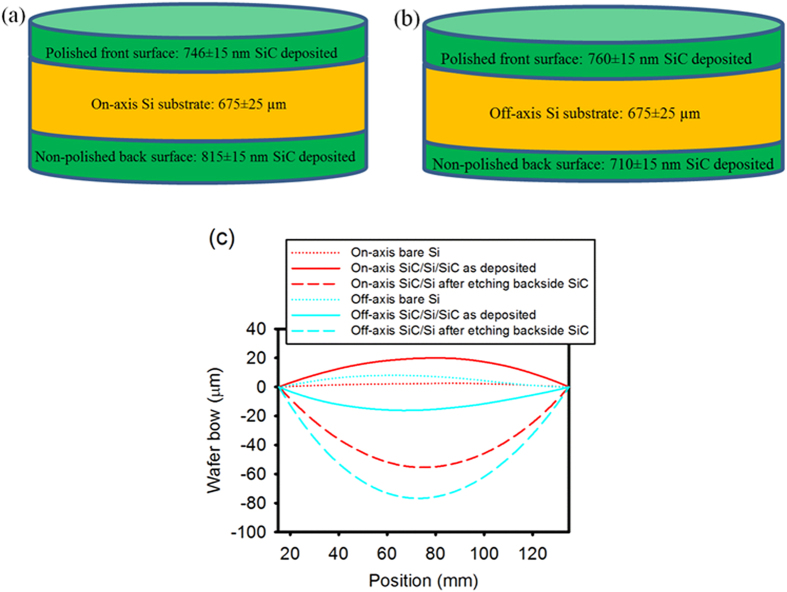
(**a**) Diagram of sandwich 3C-SiC/Si/3C-SiC structure after depositing ~750 nm (not to scale) SiC film on the on-axis Si substrate, the Si wafer’s single polished surface facing the pumping end, it has a slightly thicker (~50 nm more) SiC layer deposited at the backside, (**b**) Diagram of sandwich 3C-SiC/Si/3C-SiC structure after depositing ~750 nm (not to scale) SiC film on the off-axis Si substrate, the Si wafer’s single polished surface facing the incoming gases, it has a slightly thicker SiC layer deposited on the front side, (**c**) Wafer bow profile for bare Si substrates, as-deposited SiC/Si/SiC structures, and SiC/Si wafers after removing the SiC deposited on unpolished Si side.

In addition to surface roughness and crystal quality concerns, stress-induced cracks and wafer bow have hindered the development and application of 3C-SiC/Si(111) based technology^9^,^20^. Most of the SiC low-pressure chemical vapour deposition (LPCVD) reactors use a susceptor to hold the Si substrates, which can only deposit SiC layer on the top-side of the substrates. Moreover, the quantity of the wafers that can be processed in one run is also limited by the diameter of the susceptor, generally up to three 50-mm wafers or one 150-mm wafer^21^. For the custom-made LPCVD reactor used in this investigation, Si wafers are supported vertically in a boat thereby exposing both sides of the wafer to deposition. The schematic illustration of the reactor is similar to what is shown in ref. 22, which enables the deposition of SiC on both sides of the Si wafers. This LPCVD reactor also results in parallel/batch processing; up to seven 150-mm Si wafers can be produced in a single run with excellent thickness uniformity. A new production model of this LPCVD reactor, named Epiflx, co-developed by SPP Process Technology Systems Ltd (SPTS, a leading semiconductor equipment manufacturer) and Griffith University^23^, has demonstrated the ability to deposit SiC uniformly on 300-mm Si wafers^24^. The Epiflx system has the capacity to process up to 50, 300-mm wafers or a larger number of smaller wafers, for example, up to 150, 150-mm wafers. In this paper, wafer bow of the bare Si substrates (single-side polished, SSP) and grown 3C-SiC/Si(111) structure was measured using a Tencor Flexus 2320 system and the results are presented in Table 1 and Fig. 6. The SiC thickness uniformity across 150-mm wafer is 99.2 ± 0.5% at 1000 °C, decreasing slightly to 98.5 ± 0.8% at 1200 °C (with 10 mm edge exclusion). Curvature scans were performed along two perpendicular axes, at azimuthal angle 0°, the scan direction is parallel to the wafer flat, whereas at 90°, the scan direction is normal to the wafer flat, which enable us to investigate whether the bow and stress are orientation dependent as previously reported in the literature^12^,^25^. The bow of bare 675 ± 25 um Si wafer varied from 2.61 μm to 8.37 μm. After SiC deposition, a sandwich structure was built, where both sides of the Si substrate were coated with 750 ± 80 nm SiC film (shown in Fig. 6(a,b)). The on-axis wafer, with its single polished surface facing the pumping end, has a slightly thicker (~50 nm more) SiC layer deposited at the back (this is because the growth rate across the chamber slightly reduced with its position moving towards the pumping end), ended with a maximum convex/positive bow value of 19.98 μm (the corresponding radius of curvature is 88.90 m). In the case of the off-axis wafer, with its single polished surface facing the incoming gases, has a slightly thicker SiC layer deposited on the front side, a concave/negative bow shape (up to −16.16 μm) was observed. Due to the mismatch in lattice constant and thermal expansion coefficients, the grown SiC layer has a tensile residual stress after cooling the samples to room temperature, which is more pronounced for the thicker SiC layer, resulting in the wafer always bending towards the thicker SiC coated side. These results demonstrate that the wafer bow shape can be tuned by adjusting the SiC layer thickness deposited at the both sides of the Si substrate. The off-axis 3C-SiC/Si has smaller bow magnitude compared to the on-axis SiC/Si wafer.

After etching the SiC layer deposited on the back of Si substrate using a plasma etcher (shown in Fig. 6(c) and detailed data can be found in Table 1), the maximum bow increased from less than 20 μm to 59.79 μm and 76.75 μm for on-axis and off-axis wafers respectively. These results demonstrate that double-side SiC deposition can mediate the bow of the wafer, making it more suitable for subsequent device-related processing (According to Semi standard M1-0600, 150-mm wafers need to have a bow value of less than 60 μm to be processed in semiconductor processing equipment). The previously reported radius of curvature for 700 ~ 800 nm SiC(111) grown on 4 inch Si(111) at 1350 °C ranged from 2 m to 10 m^9^ In contrast, using our double-sided SiC coating technique, the observed minimum radius of curvature in this investigation is found to be 88.90 m and 112.27 m for ~750 nm SiC film grown on 150-mm on-axis and off-axis Si(111), respectively. Therefore, the double-side SiC deposition, performed at relatively lower temperature, minimized the wafer curvature compared to single-side SiC deposition performed at higher temperature.

It is found that the average residue stress for on-axis wafer is not azimuthal angle dependent, with an average value of 751.40 ± 1.20 MPa at a deposition temperature of 1200 °C, which is ~10% increase compared with the stress value observed at a deposition temperature of 1000 °C^12^. The residue stress for off-axis wafer varied slightly from 1077.80 MPa at 0° to 1021.70 MPa at 90° at a deposition temperature of 1200 °C, but not as strongly as it was seen at 1000 °C (737MPa at 0° and 930 MPa at 90°)^12^. However, the bow magnitude was found to be orientation dependent, and the bow magnitude for the off-axis 3C-SiC/Si wafer reduced dramatically by 50% from 16.16 μm at 0° to 7.37 μm at 90° (the bare off-axis Si wafer has very similar bow value of ~8.20 μm), meaning that the direction parallel to the step edges has a smaller wafer bow.

## Conclusions

In conclusion, step-flow dominated 3C-SiC growth is demonstrated at 1200 °C on 4° off-axis 150-mm Si substrates with a kinetic scaling exponent of zero. In contrast, 3D growth dominated deposition was observed for on-axis 3C-SiC/Si, as indicated by a large kinetic scaling factor of 0.49. Elevating the growth temperature from 1000 °C to 1200 °C enhances the surface diffusion length of the surface adatoms, and as a result, the growth mechanism changes from three dimensional island formation to step-flow mode. For ~1100 nm 3C-SiC grown on off-axis substrate, the RMS roughness is 3.99 ± 0.2 nm with a RC FWHM of 0.43°. Double-side SiC coating is demonstrated to be an effective way to minimize wafer bow, with a bow value of smaller than 20 μm compared to a value of 76.75 μm for single-side SiC deposition. Concave and convex bow shapes can be achieved *in situ* by tuning the SiC layer thicknesses deposited on the two-side of the Si substrate. The bow magnitude was found to be orientation dependent for off-axis 3C-SiC/Si wafer, reducing dramatically by 50% from 16.16 μm at 0° to 7.37 μm at 90° (relative to the wafer flat). The 3C-SiC grown on off-axis Si, compared to that grown on on-axis Si, has smaller surface roughness, narrower FWHM of SiC(111) peak, and smaller bow magnitude.

## Methods

### Growth of 3C-SiC films

3C-SiC films with thickness ranging from 10 to 1110 nm were deposited on both on-axis (off-cut angle <0.5°) and 4° off-axis (towards [110]) 150-mm Si(111) SSP substrates, using a custom-made LPCVD reactor. After removing the oxide layer from the Si substrate, using 1.0 sccm silane (SiH_4_), a carbonisation step was initiated at 750 °C to convert the top surface of Si substrate into SiC using propylene (C_3_H_6_). The epitaxial growth of SiC was performed using alternating supply epitaxy (ASE) at temperatures of 1000 °C and 1200 °C. SiH_4_ and C_3_H_6_ were used as Si-containing and C-containing precursors, respectively. For SiC films grown at 1200 °C, around 100 nm 3C-SiC layer was initially grown at 1000 °C to avoid Si void formation during the temperature ramping from 1000 °C to 1200 °C.

### Characterisation of SiC films

AFM measurements were performed using Park NX20 under non-contact mode to investigate the morphology and root mean square roughness. X-ray diffraction measurements were performed with a Panalytical Empyrean x-ray diffractometer using Cu Kα1 radiation (λ = 1.5405980 Å); it has a high resolution four-crystal Ge (220) asymmetrical incident beam monochromator and a PIXcel-3D detector with a fixed anti-scatter slit. The PIXcel detector is used in the Open Detector mode for the rocking curve measurements. Top-view surface morphology was observed using a JEOL JSM-6510LV scanning electron microscope. A Tencor Flexus 2320 system was used to measure wafer curvature before and after SiC deposition. The curvature measurements were performed along the diameter of 150-mm wafers with 15 mm edge exclusion. The biaxial stress for SiC films was calculated on the basis of the modified Stoney’s equation using the appropriate elastic moduli (170 GPa) and Poisson’s ratio (0.26) values for Si(111).

## Additional Information

**How to cite this article**: Wang, L. *et al.* Kinetic surface roughening and wafer bow control in heteroepitaxial growth of 3C-SiC on Si(111) substrates. *Sci. Rep.* **5**, 15423; doi: 10.1038/srep15423 (2015).

## Figures and Tables

**Table 1:** The radius of curvature and bow data for the bare Si substrates and deposited SiC/Si wafers.

	**Azimuthal angle**	**Bare Si**		**After SiC deposition**		**After removing backside SiC**		
		**Radius (m)**	**Bow (μm)**	**Radius (m)**	**Bow (μm)**	**Radius (m)**	**Bow (μm)**	**Stress (MPa)**
On-axis SiC/Si(111)	0	−716.25	2.61	-88.90	19.98	32.38	−55.42	752.70
	90	978.90	−2.74	−108.39	16.61	30.12	−59.79	750.20
Off-axis SiC/Si(111)	0	−258.43	8.08	112.27	−16.16	23.15	−76.75	1077.80
	90	−217.25	8.37	258.93	−7.37	24.99	−71.54	1021.70

After SiC deposition, a sandwich structure was built, where both sides of the Si substrates were coated with SiC films, then the wafer bow was measured before and after removing the backside SiC by plasma etching.
